# PSAT: A web tool to compare genomic neighborhoods of multiple prokaryotic genomes

**DOI:** 10.1186/1471-2105-9-170

**Published:** 2008-03-26

**Authors:** Christine Fong, Laurence Rohmer, Matthew Radey, Michael Wasnick, Mitchell J Brittnacher

**Affiliations:** 1 Department of Genome Sciences, University of Washington, Box 357710, Seattle, Washington 98195, USA

## Abstract

**Background:**

The conservation of gene order among prokaryotic genomes can provide valuable insight into gene function, protein interactions, or events by which genomes have evolved. Although some tools are available for visualizing and comparing the order of genes between genomes of study, few support an efficient and organized analysis between large numbers of genomes. The Prokaryotic Sequence homology Analysis Tool (PSAT) is a web tool for comparing gene neighborhoods among multiple prokaryotic genomes.

**Results:**

PSAT utilizes a database that is preloaded with gene annotation, BLAST hit results, and gene-clustering scores designed to help identify regions of conserved gene order. Researchers use the PSAT web interface to find a gene of interest in a reference genome and efficiently retrieve the sequence homologs found in other bacterial genomes. The tool generates a graphic of the genomic neighborhood surrounding the selected gene and the corresponding regions for its homologs in each comparison genome. Homologs in each region are color coded to assist users with analyzing gene order among various genomes. In contrast to common comparative analysis methods that filter sequence homolog data based on alignment score cutoffs, PSAT leverages gene context information for homologs, including those with weak alignment scores, enabling a more sensitive analysis. Features for constraining or ordering results are designed to help researchers browse results from large numbers of comparison genomes in an organized manner. PSAT has been demonstrated to be useful for helping to identify gene orthologs and potential functional gene clusters, and detecting genome modifications that may result in loss of function.

**Conclusion:**

PSAT allows researchers to investigate the order of genes within local genomic neighborhoods of multiple genomes. A PSAT web server for public use is available for performing analyses on a growing set of reference genomes through any web browser with no client side software setup or installation required. Source code is freely available to researchers interested in setting up a local version of PSAT for analysis of genomes not available through the public server. Access to the public web server and instructions for obtaining source code can be found at .

## Background

An analysis of gene order conservation is commonly performed in genomic comparison studies of microbial genomes. Several tools for visualizing and comparing gene order on a whole genome scale have been developed for identifying genomic rearrangements and to infer phylogenetic relationships between genomes [1-3]. Gene order analyses on a local genomic neighborhood level, however, can also be very useful for helping to predict gene function, identify proteins that potentially interact physically, or infer evolutionary relationships between genomes [4,5]. For example, clusters of genes conserved among several species, including distantly related species, suggest a positive selection for a particular local arrangement of genes that may indicate the existence of an operon or groups of genes that are functionally related [6-9].

Researchers frequently perform comparative genomics studies between a selected set of closely related genomes to investigate genomic differences responsible for distinct characteristics. Several tools that have been developed with features to assist researchers with comparing genomic neighborhoods between genomes therefore focus on comparisons among only a small set of genomes [10-14]. Many of these tools are also designed for local installation that requires researchers to setup software on their own workstations, and then provide sequence input files to be processed locally [10,12,14,15]. These tools are often limited in the number of genomes supported for comparison because analyses among a large number of genomes can result in long computing times and present challenges in the display of massive amount of results. The research community therefore needs tools that facilitate more efficient and manageable comparisons of genomic neighborhoods among larger sets of genomes.

We have developed the Prokaryotic Sequence homology Analysis Tool (PSAT), a web based tool that utilizes a large database of pre-calculated sequence homologs for analysis of genomic neighborhoods among large numbers of bacterial genomes. A PSAT web server is publicly available to provide researchers around the world with access to the tool's comparative analysis utilities immediately, without any software installation or setup. Several other websites have been developed with similar designs, utilizing custom databases populated with gene and homology data from multiple bacterial genomes and providing a set of analysis tools through a web interface. Some examples include MicrobesOnline, Prolinks, STRING and the TIGR Comprehensive Microbial Resource (CMR), each with its own set of comparative genomics features [16-19]. A common feature these systems all share is a graphical browser for comparing the genomic region surrounding a gene of interest with other genomes. We recognize the utility of visualization methods for studying gene context and have developed PSAT with a focus on this aspect of comparative analysis. PSAT uses an original visualization method for a sensitive gene order analysis and provides features specifically designed to facilitate comparison of genomic neighborhoods among large numbers of genomes.

## Implementation

We describe here how we generate the data to populate the database utilized by the PSAT web server. We then provide an overview of the source code developed for generating the web interface for query and display of results. Researchers interested in running a local version of PSAT can download the freely available source code and follow similar methods to create and populate their own database for studying unpublished genomes or genomes not yet added to our public PSAT tool.

Protein and sequence files for published bacterial genomes are downloaded from NCBI [20]. The protein files are parsed to populate a PostgreSQL [21] database with details about the genes including location, strand, product, and a gene index indicating positional order within the genome. Protein BLAST databases are created using sequence files for all genomes added to the database. Each genome is designated to be a reference genome or a comparison genome (or both a reference and comparison genome) and protein BLAST [22] is run for the genes of the reference genomes against the genes of each comparison genome. The top three hits for each reference gene against each comparison genome are stored in the database including details such as a gene identifier, alignment start and end, and BLAST scores such as e-value, percent identity and bit score. The tool can be extended to include any number of reference genomes, and new comparison genomes can be added as they are released to NCBI. Perl [23] scripts query the database for BLAST hits along with gene indices to determine the number of sequence homolog pairs that occur in consecutive order. This method is used to assign each BLAST hit pair a homolog cluster score that is utilized by the tool to help infer which genomes have the greatest number of genes in conserved order surrounding a given homolog pair. The homolog cluster scores are stored in the database for quick access by the tool.

Several freely available Perl modules (available from CPAN [24]) are used to generate the PSAT web interface. A web form created using the Perl CGI module collects user input for selecting genomes to compare, finding specific genes, setting homolog filters such as BLAST score thresholds, or specifying a particular type of query. The Perl DBD module is used to query the database for genes and their BLAST hits that meet the input criteria. The CGI scripts process and display the results taking into account options, such as criteria for filtering or sorting results, specified by the user. Homolog details and homolog cluster scores are formatted into an html table and by default are grouped by bacterial genus. The Perl ImageMagick module [25] is used to draw the graphic visualizing the genomic neighborhood surrounding a given gene, and the genomic neighborhoods surrounding homologs found in other genomes. Javascript is used to display gene and alignment details in popup boxes that are activated when users hover the mouse over each gene in the graphic. PSAT runs on an Apache web server [26] such that users can easily access the tool from any web browser without needing to install any additional software.

## Results and Discussion

PSAT users use the tool's web interface to select a single reference genome to perform comparisons with over five hundred bacterial genomes publicly available through NCBI. Because protein sequence homologs have been pre-computed and stored in an optimized database, retrieval of homologs among such large numbers of genomes using various querying options is quick. For the selected gene of interest and each of its homologs in the result set, PSAT generates and aligns a visualization graphic of the genomic neighborhood. Each gene in the reference genome is assigned a color, and any homolog found in the displayed region of a comparison genome is color coded to correspond with the appropriate reference gene. The coloring of homologs is designed to help researchers easily identify regions of conserved gene order across several genomes, providing support for gene orthology or functional gene clusters. To facilitate examination of gene neighborhoods, popups activated when hovering the mouse over each drawn gene provide users with gene details such as gene name, locus tag, and description, as well as any relevant BLAST hit details. A zoom tool is also available for comparing genomic regions of different sizes, ranging from 1 to 100 kb. To assist researchers with exploration of large amounts of results, features are available for scrolling through the images generated for each comparison genome to align with the reference genome, selecting to remove genomes from the visualization, or reordering the results based on BLAST hit scores or using PSAT's homolog cluster scoring system.

The PSAT homolog cluster score for a selected gene is defined as the number of contiguous homolog neighbors found in conserved order surrounding the homolog match for this gene in another genome. Homologous protein sequences are determined by BLAST alignment [22] with a user adjustable minimum alignment score threshold. Higher cluster scores suggest the existence of larger conserved gene clusters and might reflect closer phylogenetic distances between genomes, or selective pressure for gene clusters among genomes that share common properties such as a similar lifestyle. The scoring method utilized in PSAT was designed such that scores comparing large numbers of genomes can be calculated efficiently with minimal bias and preloaded within the tool for immediate access by users. The user adjustable query constraints enable researchers with varying interests to perform analyses with a range of sensitivities. Tolerant alignment score thresholds increase sensitivity in the search for gene clusters by allowing for cases where protein similarity across distantly related genomes may be relatively low. The gene context (contiguous sequence) requirement acts as a filter that significantly reduces false gene cluster predictions. This combination of factors, tolerant alignment score thresholds and contiguous genes in similar order, was found in practice to be a powerful and computationally efficient method of discovering conserved gene clusters. Among other factors that have been previously considered such as phylogenetic profile, co-occurrence in a metabolic pathway and co-occurrence in published text, conserved gene order was found to be the most determining factor for identifying conserved gene clusters [17,27]. Innovative algorithms have also been developed to identify clusters of genes whose close proximity to one another has been conserved but not necessarily the ordering of genes [8,28-31]. These approaches to modeling more complex clusters however require increased computational overhead, and adding more stringent requirements to increase accuracy can actually limit the ability to detect some conserved clusters (see for example Fulton et al. 2006 [32]). For PSAT the minimal overhead of determining the homolog cluster score enables efficient and rapid searches in a database that is not limited in size, has user adjustable alignment score thresholds, and can easily be modified to reflect alignments for updated or new genome sequences as they become available. For clusters that are interleaved with pseudogenes, local gene rearrangements, insertions, deletions or inversions the homolog cluster score will be smaller. Consequently, gene neighborhoods in closely related species that have been disrupted, and are therefore loci of potential loss of function, can easily be detected (see the second example below). Where gene rearrangements have occurred the conserved clusters of genes can be readily identified by the homolog coloring display method.

The PSAT display draws all genomic features to scale, including intergenic distances, and uses color to represent homologous genes in the compared genomes to facilitate identification of conserved gene clusters, rearrangements, insertions, deletions and sequence inversion. In the graphical display of each genome, which is aligned with the 5' end of the query gene, PSAT distinguishes the homologs with a common color (the rest of the genes are grey). For the purpose of identifying conserved gene clusters, this method of display presents some advantage over coloring genes based on role categories (see for example the Comprehensive Microbial Resource [33]) or other ontologies that represent broader concepts than sequence homologs. The genomes in PSAT are ordered by decreasing homolog cluster scores and grouped by genus. This hybrid method acknowledges the usefulness of phylogenetic information in some comparative genomic research efforts as exemplified by the MicrobesOnline "Gene Tree" display [34]. Conservation of gene clusters generally reflects conservation of function and conserved gene order suggests that the homologs in these sometimes distantly related clusters also share a similar function, and thus may be orthologs. The "mouse over" feature in PSAT provides annotation information and BLAST scores for each of the homologous sequences to enable the user to assess the evidence that might support orthology. Groups of homologs that appear in a conserved order (and to a lesser extent in the same location) across multiple genomes provide additional evidence for gene orthology. In particular, conserved gene order can strengthen very weak evidence for gene orthology, especially where these same clusters occur across several distantly related genomes. In conclusion, PSAT's unique implementation enables a sensitive analysis that can help discover orthologs through conserved gene clusters that may be missed by methods employing more stringent criteria.

Our experiences performing and assisting other researchers with comparative genomics studies inspired the development of PSAT. We were therefore able to identify multiple scenarios that we had encountered in such studies in which PSAT was or would have been helpful. Using these scenarios as examples, we demonstrate here some of the uses of PSAT and evaluate the tool's utility in assisting with particular tasks. We also discuss some of our plans for developing the tool further.

### Identification of orthologs based on conserved gene order among distant species

**Figure 1:**
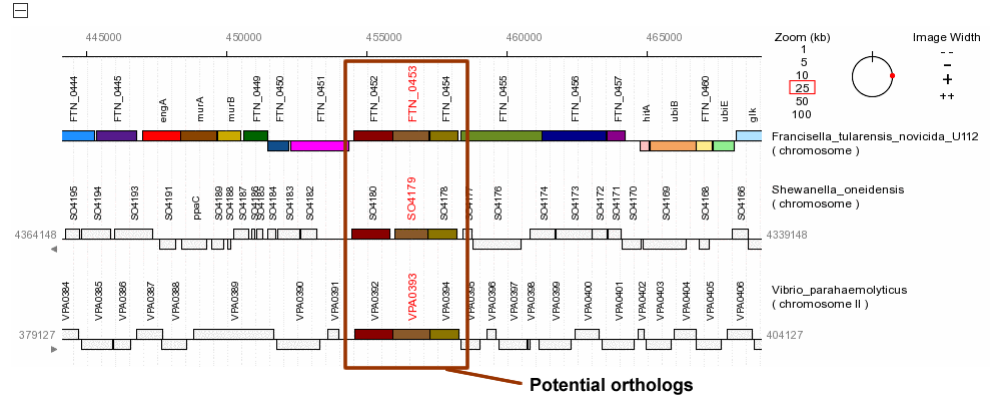
**Evidence of gene orthology through sequence homology and gene order conservation**. The *Francisella tularensis *subspecies *novicida *U112 gene *FTN_0453 *does not have any homologous genes in other *Francisella *strains. The PSAT genomic neighborhood browser, however, highlighted a gene cluster between *F t novicida *U112 and genomes from genera such as *Shewanella *and *Vibrio*. The conserved order of the homologs provided secondary evidence that the genes in this cluster are orthologs.

PSAT enables analyses that combine sequence homolog and gene context data to provide evidence of gene orthology. Researchers often use gene orthologs to assist with assigning genes a functional annotation. A genomic comparison study between closely related genomes can often identify putative orthologs for the majority of genes to be annotated. However, some genomes may not have a closely related genome that has already been annotated, or certain genes may not have an ortholog in a closely related genome. A study of the genome of *Francisella tularensis *subspecies *novicida *U112, for example, revealed that the gene *FTN_0453 *does not have any BLAST hits in the other *Francisella *genomes. PSAT results indicated, however, that the gene is homologous to genes of other bacterial genera including *Shewanella *and *Vibrio*. Although the sequence identity between *FTN_0453 *and these homologs is modest, the PSAT visualization graphic also indicated the gene may be involved in a potential gene cluster (Figure 1). Further investigation of the literature actually revealed experimental evidence suggesting the involvement of the gene cluster in *Shewanella oneidensis *with biofilm attachment and detachment [35]. This information may provide additional clues about the function of the corresponding genes in *F t novicida *U112 and the capabilities of the organism. Tools performing an analysis among a more selective set of genomes or with less sensitivity may not have discovered the potential relationship.

### Detection of a loss of function in a genome under study

**Figure 2:**
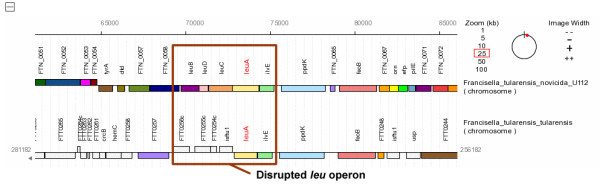
**Loss of function suggested by disruption of the *leu *operon**. The *leu *operon is known to be involved with leucine biosynthesis in *Francisella tularensis *subspecies *novicida *U112 (*ilvE*, *leuA*, *leuC*, *leuD*, and *leuB*). The PSAT graphic demonstrated that genes in this operon are missing in *Francisella tularensis *subspecies *tularensis *SchuS4 suggesting that leucine biosynthesis may be impaired in this strain of the bacteria.

Researchers can use PSAT to help detect a loss of function within particular bacterial organisms or to investigate the genetic basis of a known loss of function. When a cluster of genes is known to be involved with a specific function, an incomplete or disrupted cluster in certain genomes may suggest a loss of function. An examination of such a disruption can also provide clues to particular events which may have led to the loss. For example, a comparative study of *Francisella *genomes involved investigation of the conservation of the *leu *operon. This operon, which includes the genes *ilvE*, *leuA*, *leuC*, *leuD*, and *leuB*, is known to be involved with leucine biosynthesis in *Francisella tularensis *subspecies *novicida *U112. Performing a PSAT analysis for these genes indicated that this cluster is incomplete in the *Francisella tularensis *subspecies *tularensis *SchuS4 strain, with an absence of *leuC*, *leuD*, and *leuB*, suggesting that leucine biosynthesis is impaired in this strain of the bacteria (Figure 2) [36]. Further investigation into this interesting observation suggested specific IS element insertion events involved in the inactivation and provided critical clues into the evolutionary history of various strains of the bacteria [36].

### Investigation of biological association between genes

**Figure 3:**
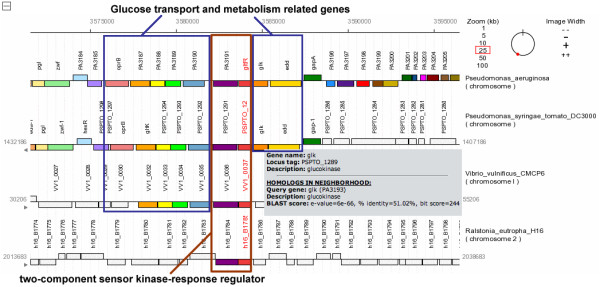
**Genes related to glucose metabolism neighbor a pair of regulator genes**. *Pseudomonas aeruginosa *genes *gltR *and *gltS *make up a putative sensor kinase-response regulator pair. PSAT demonstrated that the gene pair is located near a cluster of genes involved with glucose metabolism in several genomes. Previous studies have shown that *gltR *is indeed required for glucose transport in *Pseudomonas aeruginosa*.

An analysis of gene context across multiple genomes, including those of distantly related species, can be useful for an investigation of biological association between genes. For example, PSAT enabled an exploration of the genomic neighborhood of a putative sensor kinase-response regulator (SK-RR) pair in *Pseudomonas aeruginosa*, coded by genes *gltR *and *gltS*. SK-RR gene pairs are typically located next to each other and are also sometimes located next to genes for which they modulate transcription. PSAT revealed how the SK-RR pair is conserved across genomes, including those from distantly related species. In addition, the results show how the SK-RR pair appears in a cluster of genes related to glucose metabolism in many of these genomes (Figure 3). The observation that these glucose transport and metabolism related genes are frequently located in the neighborhood of gltR-gltS suggests that their regulation is controlled by the SK-RR. These results effectively reflect past research showing that *gltR *is needed to activate some of the genes related to glucose transport and metabolism [37].

### Future Development

The PSAT web server's database of gene annotations and sequence homologs enables the tool's efficient genomic neighborhood comparative analysis and visualization features. The public server is currently loaded with a selected set of reference bacterial genomes published through NCBI. We plan to continue to load additional published genomes into the tool in order to more broadly support researchers studying different bacterial organisms. We also recognize the importance of keeping the genome data used by the public server up to date. We are therefore also implementing an automated system for updating the database as new or modified genome annotations become available through NCBI.

PSAT was originally designed for exploring homologs within genomic neighborhoods to analyze gene order and identify potential gene clusters among several genomes. We recognize, however, that the database of genes and protein sequence homologs that was built for this purpose could be utilized for several other types of genomic analyses. We plan to, for example, leverage the database to add a query interface to retrieve homology statistics from various genomes. Enabling researchers to determine the proportion of genes in a reference genome that have homologs in several other genomes can provide a rough comparison of genomic similarity and phylogenetic distances. Another feature we plan to implement is a querying method utilizing homology data for determining a putative set of genes that are unique to a particular set of genomes. This kind of analysis can help researchers identify a list of potential genes that appear, for example, in a set of genomes belonging to virulent strains of a bacterium, yet not in a set of genomes belonging to avirulent strains of the same bacterium.

## Conclusion

As the number of bacterial genomes being sequenced, annotated, and published quickly increases, so does the potential for researchers to perform interesting comparative studies based on the available genomic data. The PSAT web tool can be helpful for such comparative genomics studies by providing researchers with an original interface to explore and compare the genomic neighborhoods of multiple prokaryotic genomes. Essential features of the tool include efficient retrieval of homologs between large numbers of genomes, a graphical visualization of homologs within genomic neighborhoods for analyzing gene order conservation, and options for ordering and filtering results based on various properties to facilitate exploration of large result sets. We have demonstrated how PSAT can be used to help identify gene orthologs, detect a loss of function in a genome under study, and discover potential biological associations between genes.

Our publicly available PSAT web server currently supports analysis of reference genomes from a subset of published bacterial genomes, including selected genomes from the *Burkholderia, Escherichia, Francisella*, *Salmonella*, *Pseudomonas*, and *Yersinia *genera, against over five hundred other bacterial genomes available on NCBI. The PSAT source code is also freely available for researchers to easily set up local versions of PSAT to perform analyses of other genomes, including those not yet released to the public. Please visit the PSAT website for more information.

## Availability and requirements

**Project name**: PSAT

**Project home page**: 

**Operating systems**: Platform independent for accessing the public web server; Linux (or possibly other Unix variants) for local installations

**Programming language**: Perl

**Other requirements**: Javascript enabled web browser for accessing the public web server; Apache web server and PostgreSQL database for local installations

**Any restrictions to use by non-academics**: none

## Authors' contributions

CF designed and wrote the application, and drafted the manuscript. LR advised on biological aspects of the application and helped draft the manuscript. MW and MR assisted in the computational architecture design. MB conceived of the application, coordinated its design and helped to draft the manuscript. All authors read and approved the final manuscript.
